# Supplementary material for: A transthalamic pathway crucial for perception
Source: Nat Commun. 2024 Jul 26;15:6300. doi: 10.1038/s41467-024-50163-w (PMC11282105; doi:10.1038/s41467-024-50163-w)
Supplement: Supplementary file 1 — Supplementary Information [file 41467_2024_50163_MOESM1_ESM.pdf]

Supplementary Information

Supplementary Methods

ANATOMICAL VERIFICATION OF THE S1 L5 TO POM TO S2 PATHWAY

With regards to **Supplementary Fig. 1**, Rbp4-Cre mice were injected with Cre-dependent Jaws-TdTomato and implanted with a head bar and cranial window above S1 and S2, as described in **Surgical procedures**. Intrinsic signal optical imaging was performed to identify S1 and S2 (see **Intrinsic signal optical imaging**) and 80nl of the retrograde tracer, FluoroGold, was injected into S2. One week later, the mice were perfused and sectioned (50μm thick) to locate the region of POm with overlapping Jaws-TdTomato and Fluorogold cell body expression.

IN VIVO ELECTROPHYSIOLOGY

With regards to **Supplementary Fig. 2**, we validated Jaws opsin inhibition at S1 L5 to POm terminals. Jaws and ChR2 were expressed in S1 L5 neurons by injecting 450nl of a 1:1 mix of AAV8-CAG-FLEX-Jaws-KGC-TdTomato-ER2 and AAV5-DIO-ChR2-eYFP (UNC Vector Core) in Rbp4-Cre mice (n=4). After 3 - 4 weeks, mice were anesthetized with urethane (1.3mg/g), secured in a stereotaxic frame and a craniotomy (6mm x 5mm) was made over S1 and POm. A 470nm LED (M470L3, Thorlabs) connected to a 200μm, 0.5NA patch cable with bare fiber tip (Plexon) was placed just above exposed S1 (ML: 3.1mm, AP: -0.8) and multiunit recordings were made in POm (DV: −3.1, ML: +1.3, AP: −1.4 mm) using custom pulled tungsten-in-glass microelectrodes (1Mohm). The microelectrode was secured alongside a patch cable with an optic fiber stub (200μm diameter, 0.5NA, Thorlabs) such that the tip of the electrode was a maximum distance of 290μm from the tip of the optic fiber. The electrode and optic fiber were lowered into POm using a stereotaxic arm. The patch cable was connected to a 620nm LED (maximum 137mW/mm^2, PlexBright, Plexon). A data acquisition device (NI USB-6009) and custom Matlab code coordinated 470nm LED pulses (7 per trial, 1Hz, 20msec long) and 620nm LED pulses (1 per trial, 1-3sec long) with various relative onset times (50msec, 500msec, 1sec, 2sec) (See **Fig. 1B**). Signals were passed through a 300Hz high-pass and 12000Hz low-pass second-order Butterworth filter. The window for spike analysis was 6-40msec from the start of the 470nm pulse and spikes were thresholded to 5 standard deviations above baseline. Responses to 470nm-only pulses were averaged for each trial and trials were pooled across electrode positions and mice. For Jaws LED offset analyses, the response to the 470nm-only pulse prior to 620nm LED onset was compared to the response to the 470nm-only pulse immediately after 620nm LED offset.

MULTIPLE LINEAR REGRESSION ANALYSIS

With regards to **Supplementary Fig. 6**, each neuron's responses were regressed against two categorical task variables: texture stimulus (G5 or G0) and go/no-go lick choice of the mouse. The linear regression model was fitted with the fitlm function in MATLAB. Formally,

$$Y = \beta_0 + \beta_{texture}\, x_{texture} + \beta_{lick}\, x_{lick} + \varepsilon$$

where Y is the calcium fluorescence activity during the sensory epoch (sensory AUC minus baseline AUC of the dF/F trace), β_0 is the intercept of the regression line, $\beta_{texture}$ is the regression coefficient for stimulus texture $x_{texture}$ (1=G5 and 0=G0), β_{lick} is the regression coefficient for lick choice x_{lick} (1=lick and 0=no-lick), and ε estimates the residual. The statistical significance of each regressor was determined using permutation testing, in which a shuffled population distribution was generated by shuffling the response variable and recalculating the regression coefficients (repeated 1,000 times). The significance level was set at p<0.05 above shuffled conditions.

Supplementary Tables

Supplementary Table 1. Statistical analysis of data

Figure	Data (mean ± sem)	P-value	Statistical test
**Fig. 2B**	**Error rate No-laser vs Sensory laser**<br>0.23 ± 0.013 vs 0.41 ± 0.023	 p=0.0039	Paired samples Wilcoxon test
**Fig. 2C**	**Psychometric curve No-laser vs Sensory laser**<br>effect of texture<br>effect of laser<br>texture x laser interaction<br>*No-laser vs Sensory laser*<br>G0: 26.54 ± 2.14 vs 44.44 ± 3.67<br>G1: 43.29 ± 2.60 vs 45.64 ± 3.28<br>G2: 54.67 ± 3.69 vs 53.78 ± 3.03<br>G3: 74.44 ± 3.68 vs 64.14 ± 5.58<br>G4: 74.16 ± 3.56 vs 60.73 ± 4.84<br>G5: 79.17 ± 2.52 vs 63.20 ± 4.35	 p<0.0001<br>p=0.094<br>p<0.0001<br><br>p=0.0078<br>p>0.99<br>p>0.99<br>p=0.18<br>p=0.017<br>p=0.0016	2-way RM ANOVA<br><br>Bonferroni post-hoc tests
	**Psychometric parameters No-laser vs Sensory laser**<br>lapse rate: 0.22 ± 0.031 vs 0.37 ± 0.044<br>guess rate: 0.20 ± 0.041 vs 0.40 ± 0.039<br>bias: 2.51 ± 0.23 vs 2.49 ± 0.33<br>sensitivity: 1.11 ± 0.20 vs 0.11 ± 0.10	 p=0.0039<br>p=0.0078<br>p=0.82<br>p=0.0039	Paired samples Wilcoxon test
**Fig. 2C**	**D-prime No-laser vs Sensory laser**<br>effect of texture<br>effect of laser<br>texture x laser interaction<br>*No-laser vs Sensory laser*<br>G1: 0.47 ± 0.035 vs 0.029 ± 0.072<br>G2: 0.76 ± 0.085 vs 0.24 ± 0.076<br>G3: 1.33 ± 0.10 vs 0.54 ± 0.17<br>G4: 1.32 ± 0.12 vs 0.43 ± 0.15	 p<0.0001<br>p<0.0001<br>p=0.0007<br><br>p=0.0012<br>p=0.0065<br>p=0.0012<br>p=0.0015	Two-way repeated measures (RM) ANOVA<br>Bonferroni post-hoc tests

	G5: 1.48 ± 0.087 vs 0.50 ± 0.12	p<0.0001	
**Fig. 2D**	**D-prime performance across trial conditions**<br>effect of condition<br>No-laser vs Sensory laser: 1.43 ± 0.080 vs 0.50 ± 0.12<br>No-laser v Delay laser: 1.43 ± 0.080 vs 0.95 ± 0.13<br>No-laser vs No whiskers: 1.43 ± 0.080 vs 0.28 ± 0.071	 p<0.0001<br>p<0.0001<br>p=0.025<br>p=0.025	Mixed-effects model Bonferroni post-hoc tests
**Fig. 2E**	**Error rate No-laser vs Delay laser**<br>0.25 ± 0.017 vs 0.32 ± 0.023	 p=0.0055	Paired samples Wilcoxon test
**Fig. 2F**	**Psychometric curve No-laser vs Delay laser**<br>effect of texture<br>effect of laser<br>texture x laser interaction<br>*No-laser vs Delay laser*<br>G0: 29.14 ± 1.91 vs 35.06 ± 2.41<br>G1: 41.04 ± 1.80 vs 38.69 ± 2.84<br>G2: 62.26 ± 2.36 vs 50.92 ± 3.14<br>G3: 71.58 ± 2.25 vs 66.84 ± 3.72<br>G4: 73.84 ± 1.70 vs 70.09 ± 2.80<br>G5: 78.82 ± 2.11 vs 70.59 ± 3.18	 p<0.0001<br>p=0.0098<br><br>p=0.027<br>p=0.055<br>p>0.99<br>p=0.23<br>p=0.99<br>p>0.99<br>p=0.35	2-way RM ANOVA<br><br><br><br>Bonferroni post-hoc tests
	**Psychometric parameters No-laser vs Delay laser**<br>lapse rate: 0.24 ± 0.016 vs 0.30 ± 0.027<br>guess rate: 0.30 ± 0.032 vs 0.37 ± 0.030<br>bias: 2.73 ± 0.13 vs 3.35 ± 0.14<br>sensitivity: 0.057 ± 0.29 vs 0.0036 ± 0.13	 p=0.027<br>p=0.016<br>p=0.039<br>p=0.91	Paired samples Wilcoxon test
**Fig. 2F**	**D-prime No-laser vs Delay laser**<br>effect of texture<br>effect of laser<br>texture x laser interaction<br>*No-laser vs Delay laser*<br>G1: 0.33 ± 0.056 vs 0.097 ± 0.063<br>G2: 0.87 ± 0.083 vs 0.41 ± 0.11<br>G3: 1.14 ± 0.092 vs 0.85 ± 0.14<br>G4: 1.2 ± 0.084 vs 0.93 ± 0.10<br>G5: 1.38 ± 0.11 vs 0.95 ± 0.13	 p<0.0001<br>p=0.0011<br>p=0.36<br><br>p=0.023<br>p=0.048<br>p=0.10<br>p=0.029<br>p=0.11	 Two-way RM ANOVA Bonferroni post-hoc tests
**Fig. 2G**	**POm implant No-laser vs Sensory vs Delay laser**<br>effect of texture<br>effect of laser<br>texture x laser interaction<br>*No-laser vs Sensory laser*<br>G0: 0.30 ± 0.024 vs 0.43 ± 0.027<br>G1: 0.45 ± 0.029 vs 0.49 ± 0.026<br>G2: 0.70 ± 0.034 vs 0.60 ± 0.039<br>G5: 0.80 ± 0.015 vs 0.66 ± 0.041	 p<0.0001<br>p=0.12<br>p<0.0001<br><br>p=0.0007<br>p=0.90<br>p=0.15<br>p=0.0086	Two-way RM ANOVA<br><br><br><br>Bonferroni post-hoc tests

	No-laser vs Delay laser<br>G0: 0.30 ± 0.024 vs 0.38 ± 0.029<br>G1: 0.45 ± 0.029 vs 0.46 ± 0.023<br>G2: 0.70 ± 0.034 vs 0.55 ± 0.029<br>G5: 0.80 ± 0.015 vs 0.72 ± 0.030<br><br>**Control implant**<br>effect of texture<br>effect of laser<br>texture x laser interaction<br>*No-laser vs Sensory laser*<br>G0: 0.34 ± 0.042 vs 0.40 ± 0.023<br>G1: 0.43 ± 0.044 vs 0.44 ± 0.032<br>G2: 0.67 ± 0.050 vs 0.67 ± 0.039<br>G5: 0.76 ± 0.026 vs 0.74 ± 0.035<br>No-laser vs Delay laser<br>G0: 0.34 ± 0.042 vs 0.31 ± 0.064<br>G1: 0.43 ± 0.044 vs 0.46 ± 0.053<br>G2: 0.67 ± 0.050 vs 0.60 ± 0.060<br>G5: 0.76 ± 0.026 vs 0.72 ± 0.057	 p=0.14<br>p>0.99<br>p=0.0047<br>p=0.040<br><br><br>p=0.0027<br>p=0.16<br>p=0.43<br><br>p=0.91<br>p<0.999<br>p>0.999<br>p=0.72<br><br>p>0.999<br>p>0.999<br>p=0.26<br>p>0.999	Two-way RM ANOVA<br><br>Bonferroni post-hoc tests
**Fig. 2H**	**Jaws and behavior correlation**<br>r: 0.73<br><br>R-squared: 0.51	 p=0.030<br><br>p=0.030	 Pearson's correlation<br>Simple linear regression
**Fig. 2I**	**Whisking**<br>effect of trial<br>effect of laser<br>trial x laser interaction	 p=0.91<br>p=0.58<br>p=0.55	Two-way ANOVA
**Fig. 3D**	**Detection parameters Jaws No-laser vs Laser**<br>lapse rate: 0.17 ± 0.044 vs 0.20 ± 0.039<br>guess rate: 0.28 ± 0.47 vs 0.28 ± 0.047<br>bias: 4.21 ± 0.44 vs 5.02 ± 0.28<br>sensitivity: 1.10 ± 0.58 vs 0.69 ± 0.27	 p=0.22<br>p=0.91<br>p=0.048<br>p=0.49	Paired t-test
**Fig. 3F**	**Detection parameters No-Jaws No-laser vs laser**<br>lapse rate: 0.21 ± 0.072 vs 0.19 ± 0.028<br>guess rate: 0.27 ± 0.021 vs 0.28 ± 0.026<br>bias: 4.20 ± 0.32 vs 4.50 ± 0.15<br>sensitivity: 0.88 ± 0.39 vs 0.91 ± 0.26	 p=0.83<br>p=0.77<br>p=0.47<br>p=0.96	Paired t-test
**Fig. 4A**	**Behavioral performance of 2-photon imaging mice**<br>effect of texture<br>effect of sensory laser<br>texture x laser interaction<br>*No-laser vs Sensory laser*<br>G0: 0.23 ± 0.03 vs 0.43 ± 0.048	 p<0.0001<br>p=0.6958<br>p=0.0001<br><br>p=0.0071	Two-way RM ANOVA

	G1: 0.45 ± 0.048 vs 0.51 ± 0.052<br>G2: 0.58 ± 0.054 vs 0.51 ± 0.054<br>G5: 0.70 ± 0.02 vs 0.56 ±0.025	p>0.099<br>p=0.76<br>p=0.0002	Bonferroni post-hoc tests
**Fig. 4C**	**Overall texture responsiveness**<br>effect of brain region<br>effect of laser<br>brain region x laser interaction	 p=0.88<br>p=0.16<br>p=0.60	Two-way RM ANOVA
**Fig. 4D**	**Texture responsiveness: hit vs CR**<br>*S1*<br>effect of laser<br>effect of texture<br>laser x texture interaction<br>*S2*<br>effect of laser<br>effect of texture<br>laser x texture interaction<br>No-laser: G5 Hit vs. No-laser:G0 CR<br>No-laser: G5 Hit vs. Laser:G5 Hit<br>No-laser: G5 Hit vs. Laser:G0 CR<br>No-laser: G0 CR vs. Laser:G5 Hit<br>No-laser: G0 CR vs. Laser:G0 CR<br>Laser: G5 Hit vs. Laser:G0 CR	 <br>p=0.56<br>p=0.0033<br>p=0.91<br><br>p=0.64<br>p=0.31<br>p=0.0002<br>p=0.0013<br>p=0.032<br>p>0.99<br>p>0.99<br>p=0.68<br>p=0.86	 <br>Two-way<br>RM<br>ANOVA<br><br><br><br><br>Bonferroni<br>post-hoc<br>tests
**Fig. 4E**	**Scatterplots of hit vs CR responsiveness**<br>*S1 no-laser vs laser*<br>No-laser r=0.69<br>Laser r=0.65<br>No-laser R-squared: 0.48<br>Laser R-squared: 0.43<br>S1 Slopes different<br>*S2 no-laser vs laser*<br>No-laser r=0.72<br>Laser r=0.77<br>No-laser R-squared: 0.52<br>Laser R-squared: 0.59<br>S2 Slopes different<br><br>**Scatterplots of hit vs FA responsiveness**<br>*S1 no-laser vs laser*<br>No-laser r=0.63<br>Laser r=0.69<br>No-laser R-squared: 0.39<br>Laser R-squared: 0.48<br>S1 Slopes different<br>*S2 no-laser vs laser*<br>No-laser r=0.51<br>Laser r=0.61	 <br>p<0.0001<br>p<0.0001<br>p<0.0001<br>p<0.0001<br>p=0.24<br><br>p<0.0001<br>p<0.0001<br>p<0.0001<br>p<0.0001<br>p<0.0001<br><br><br><br>p<0.0001<br>p<0.0001<br>p<0.0001<br>p<0.0001<br>p=0.59<br><br>p<0.0001<br>p<0.0001	 <br>Pearson's<br>correlation<br>Simple<br>linear<br>regression<br><br>Pearson's<br>correlation<br>Simple<br>linear<br>regression<br><br><br><br>Pearson's<br>correlation<br>Simple<br>linear<br>regression<br><br>Pearson's<br>correlation

	No-laser R-squared: 0.26<br>Laser R-squared: 0.37<br>S2 Slopes different	p<0.0001<br>p<0.0001<br>p=0.0002	Simple linear regression
**Fig. 4F**	**Fraction positive or negative DI cells**<br>*S1*<br>no-laser G5: 0.064 ± 0.016 vs no-laser G0: 0.019 ± 0.0090<br>laser G5: 0.03 ± 0.012 vs laser G0: 0.034 ± 0.018<br>no-laser G5: 0.064 ± 0.016 vs laser G5: 0.03 ± 0.012<br>*S2*<br>no-laser G5: 0.084 ± 0.017 vs no-laser G0: 0 ± 0<br>laser G5: 0.021 ± 0.014 vs laser G0: 0.072 ± 0.024<br>no-laser G5: 0.084 ± 0.017 vs laser G5: 0.021 ± 0.014<br>no-laser G0: 0 ± 0 vs laser G0: 0.072 ± 0.024	 <br>p=0.016<br>p=0.27<br>p=0.051^<br><br>p=0.0009<br>p=0.18<br>p=0.025^<br>p=0.0013^	Paired mutually exclusive McNemar's test<br>^ z-test
	**Total fraction selective DI cells**<br>*No-laser vs laser*<br>S1: 0.082 ± 0.018 vs 0.063 ± 0.038<br>S2: 0.084 ± 0.017 vs 0.080 ± 0.022	 <br>p=0.62<br>p=0.87	Wilcoxon matched-pairs signed rank test

Supplementary Figures

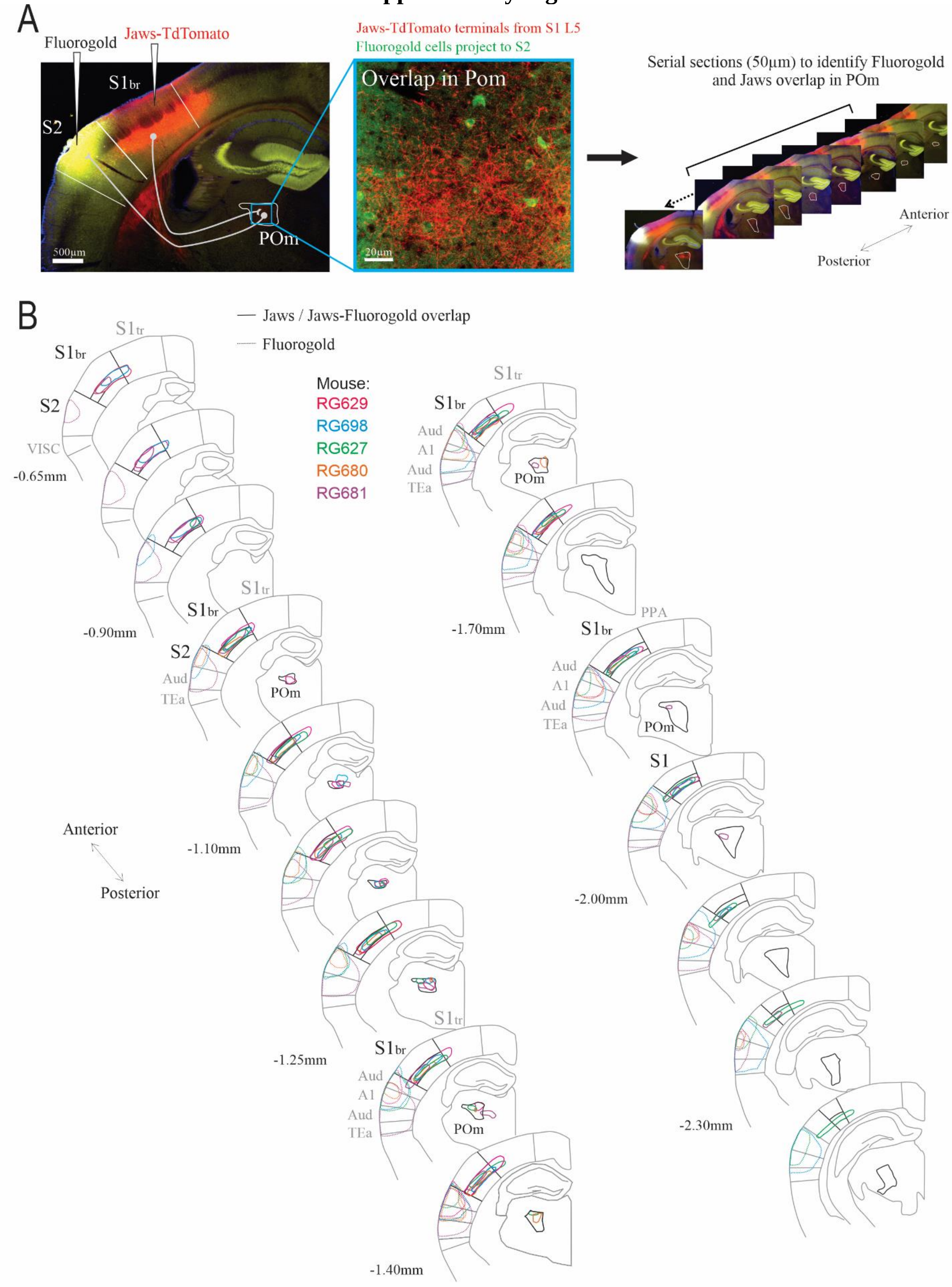

Supplementary Figure 1. Anatomical verification of the S1 L5 to POm to S2 projection

(**A**) (Left) Injection strategy to label the feedforward transthalamic pathway: Jaws-TdTomato injected in S1 L5 and retrograde tracer Fluorogold injected in the upper layers of S2 in Rbp4-Cre mice. S2 was located by functional (intrinsic signal optical) imaging. (Middle) Retrograde expression of Fluorogold cell bodies (pseduocolored green) with overlapping S1 L5 terminals in POm. (Right) Sectioning to identify Jaws and Fluorogold overlay in POm.

(**B**) Coronal sections of Jaws-TdTomato and Fluorogold (dotted outlines) expression. Each color represents one experiment per mouse. POm is outlined in thalamus. Note the overlapping area in POm is in the anterior half of the nucleus. Approximate anterior-posterior coordinates relative to Bregma are indicated.

Aud: auditory area, A1: primary auditory cortex, POm: posterior medial nucleus, PPA: posterior parietal association areas, S1: primary somatosensory cortex, S1tr: trunk area of S1, S1br: barrel field (whisker area) of S1, S2: secondary somatosensory cortex, TEa: Temporal association areas, VISC: Visceral area. Brain sections traced from the Allen Mouse Brain Atlas, https://atlas.brain-map.org/

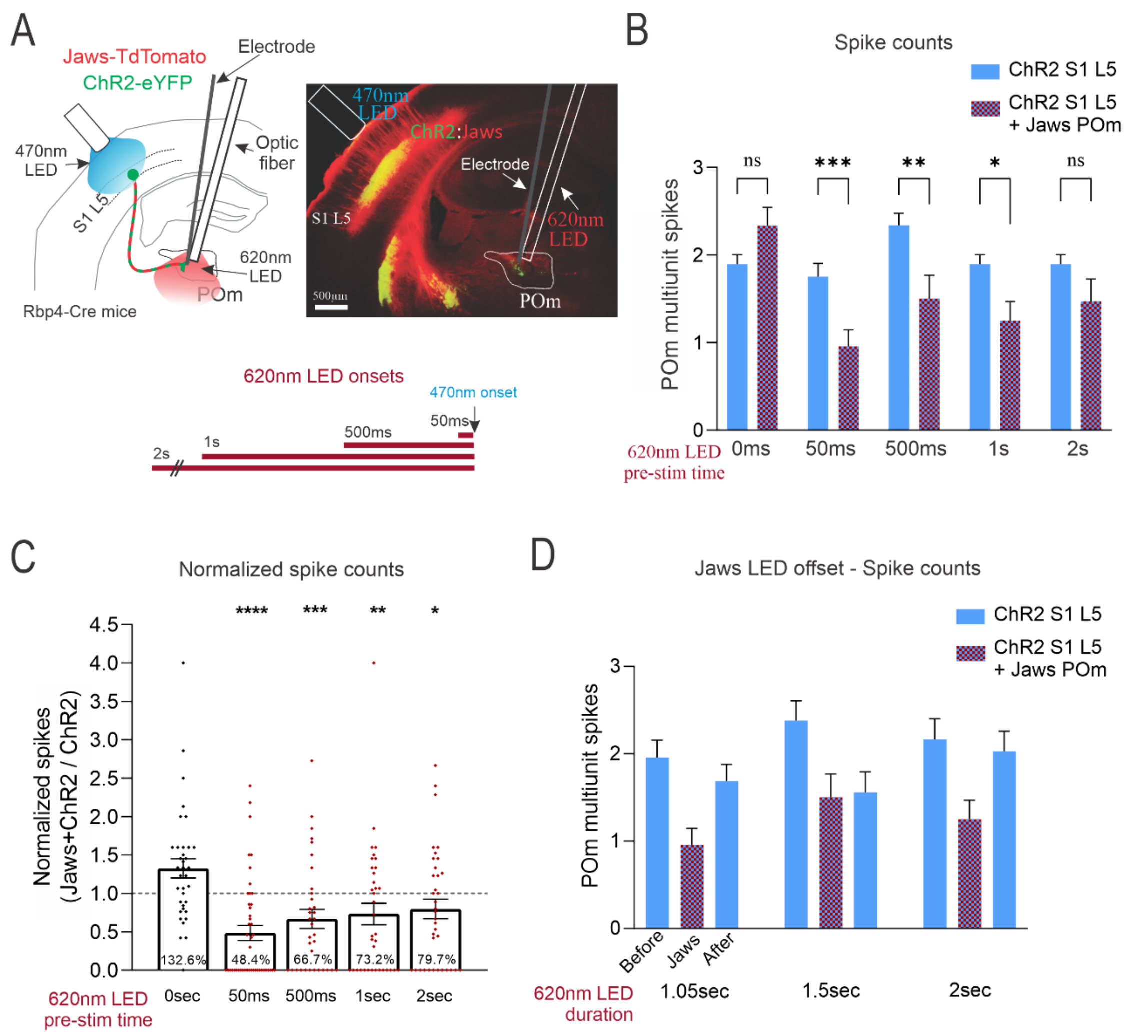

Supplementary Figure 2. Verification of Jaws inhibition in the S1 L5 to POm projection
(**A**) (Top left) Schematic of strategy to express ChR2 and Jaws in S1 L5 neurons and stimulate the projection using a 470nm LED above S1 in urethane-anesthetized mice. The output was recorded in POm terminals and Jaws inhibition tested by applying a 620nm LED attached to the electrode. (Top right) An example of opsin expression and electrode placement verified post-hoc. (Bottom) Schematic of Jaws LED application onset times relevant to a ChR2 LED activation pulse (20msec long).
(**B**) Averaged number of multiunit POm spikes in response to ChR2 stimulation of S1 L5 (blue bars) with corresponding Jaws inhibition of POm terminals (blue-red bars) at various onsets noted on the x-axis. Spikes were defined as unit activity 5 standard deviations above baseline during the 6 - 40msec time window after ChR2 onset. Two-way RM ANOVA: Opsin x onset interaction p=0.0005, Bonferroni post-hoc tests: 0msec: p=0.29, 50msec: 0.0007, 500msec: 0.0025, 1sec: 0.027, 2sec: 0.033.
(**C**) Multiunit spikes in POm during 620nm and 470nm LED application, normalized by 470nm LED -only stimulation. Mean normalized percentage is indicated on the bars. One-way ANOVA with Kruskal–Wallis post-hoc test: 0 sec vs 50 msec: p<0.0001, vs 500 msec: p=0.001, vs 1 sec: p=0.002, vs 2 sec: p=0.011.

(**D**) Averaged POm spike counts in response to S1 L5 ChR2 stimulation before, during and immediately after the 620nm LED was applied. S1 L5-induced POm activity did not change at the offset of the Jaws LED. Two-way RM ANOVA: Effect of Opsin p=0.013, Opsin x 620nm duration p=0.21, Effect of 620nm duration p=0.49.
Data are from 34-45 trials per time point, 9 experiments, 4 mice. Values shown as mean ± standard error of the mean. Note that not all terminals which express ChR2 will co-express Jaws so the magnitude of suppression is likely an underestimate. Brain sections traced from the Allen Mouse Brain Atlas, https://atlas.brain-map.org/

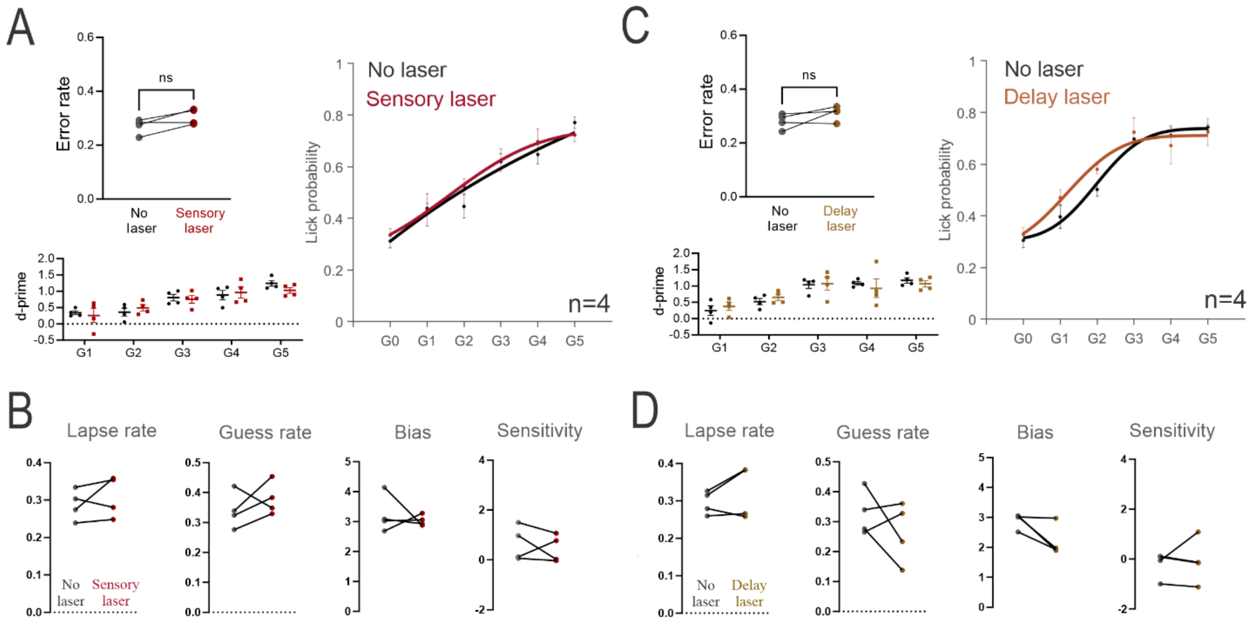

Supplementary Figure 3. Behavioral performance of TdTomato-expressing mice during a texture discrimination task

(**A**) (Top left) Error rate for no-laser and sensory laser conditions calculated from the average rate of Miss and FA trials, for G5 and G0 discrimination (0.27 ± 0.014 vs 0.31 ± 0.015, p=0.25, paired samples Wilcoxon test, n=4 mice). (Bottom left) D-prime performance across all textures for no-laser (black) vs sensory laser (red). Two-way RM ANOVA: effect of texture p=0.0047, effect of laser p=0.85, texture x laser interaction p=0.37. (Right) Psychometric curve fits for averaged data. Data points from 4 mice were an average of 5 sessions each.

(**B**) Psychometric parameters quantified from curve in A. No-laser (black) vs sensory laser (red) trials for lapse rate: 0.29 ± 0.02 vs 0.31 ± 0.027, p=0.62, guess rate: 0.34 ± 0.030 vs 0.38 ± 0.027, p=0.62, bias: 3.23 ± 0.31 vs 3.04 ± 0.091, p=0.87, sensitivity: 0.66 ± 0.35 vs 0.46 ± 0.27, p=0.62 (n=4 mice, paired samples Wilcoxon test).

(**C**) (Top left) Error rate for no-laser (black) and delay laser (brown) trials. No-laser 0.28 ± 0.014 vs delay laser 0.31 ± 0.014, p=0.25, paired samples Wilcoxon test. (Bottom left) D-prime performance for no-laser vs delay laser. Two-way RM ANOVA: effect of texture p=0.0099, effect of laser p=0.97, texture x laser interaction p=0.46. (Right) Psychometric curve fits for averaged data.

Data are averaged from the same 4 mice tested in sensory laser experiments (4-6 sessions per mouse).

(**D**) Psychometric parameters quantified from curve in C. No-laser (black) vs delay laser (brown) trials for lapse rate: 0.20 ± 0.015 vs 0.32 ± 0.035, p=0.37, guess rate: 0.33 ± 0.037 vs 0.26 ± 0.050, p=0.62, bias: 2.90 ± 0.13 vs 2.20 ± 0.26, p=0.12, sensitivity: -0.22 ± 0.26 vs -0.078 ± 0.45, p=0.87 (paired samples Wilcoxon test, n=4 mice).

Values shown as mean ± standard error of the mean.

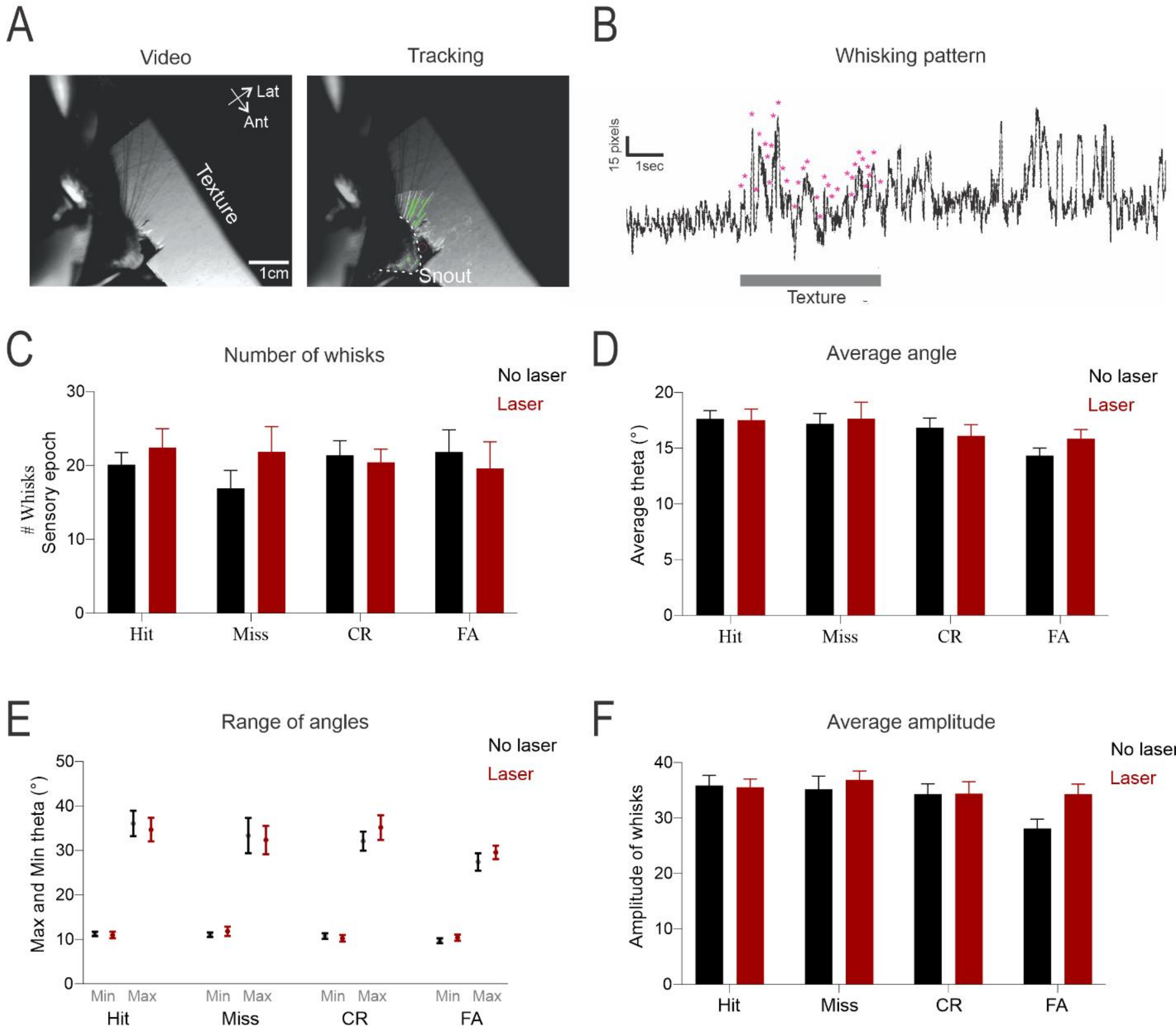

Supplementary Figure 4. Whisking

(**A**) (Left) Frame of video recorded from above during behavioral task and 2-photon imaging. (Right) Same frame with overlaid Hough transform lines (green) during whisker tracking. Tracked lines were averaged to give whisking output for each frame.

(**B**) Raw whisking pattern output of tracking based on Hough transform (see **Methods**). Due to averaging tracked whiskers for each frame, the whisking pattern showed small displacement jitters even when the whiskers were not moving. Therefore, a minimum threshold of displacement was applied for each experiment, based on visual confirmation of whisker movements across sample videos. The thresholds were 25 pixels for n=3 experiments and 15 pixels for n=1. Whisking parameters during the sensory epoch of the task were calculated based on the whisker displacements that were above the threshold (asterisks).

(**C**) Number of whisks during the sensory epoch of the task for no-laser (black) and laser (red) trials. Two-way ANOVA, effect of laser p=0.58, effect of trial type p=0.91, laser x trial type interaction p=0.55.

(**D**) Averaged theta angles during the sensory epoch for no-laser (black) and laser (red) trials. Two-way ANOVA, effect of laser p=0.69, effect of trial type p=0.057, laser x trial type interaction p=0.71.
(**E**) Maximum and minimum theta angles during the sensory epoch for no-laser (black) and laser (red) trials. Maximum and minimum data are presented on the same graph but analyzed separately by 2-way ANOVA. Maximum: Effect of laser p=0.72, effect of trial type p=0.095, laser x trial type interaction p=0.80. Minimum: Effect of laser p=0.74, effect of trial type p=0.27, laser x trial type interaction p=0.77.
(**F**) Average amplitude of whisks identified in the whisking pattern during the sensory epoch estimated by displacement of the whisking pattern from trough to peak, for no-laser (black) and laser (red) trials. Two-way ANOVA, effect of laser p=0.18, effect of trial type p=0.086, laser x trial type interaction p=0.35.
Data are from 4 experiments from 2 imaged mice (Hit trials: n=25, Hit laser trials: n=19, miss trials: n=12, miss laser trials: n=14, CR trials: n=26, CR laser trials: n=14, FA trials: n=13, FA laser trials: n=15). Note that delay laser, G2 and G1 trials during the same behavioral sessions are not shown. Values shown as mean ± standard error of the mean.

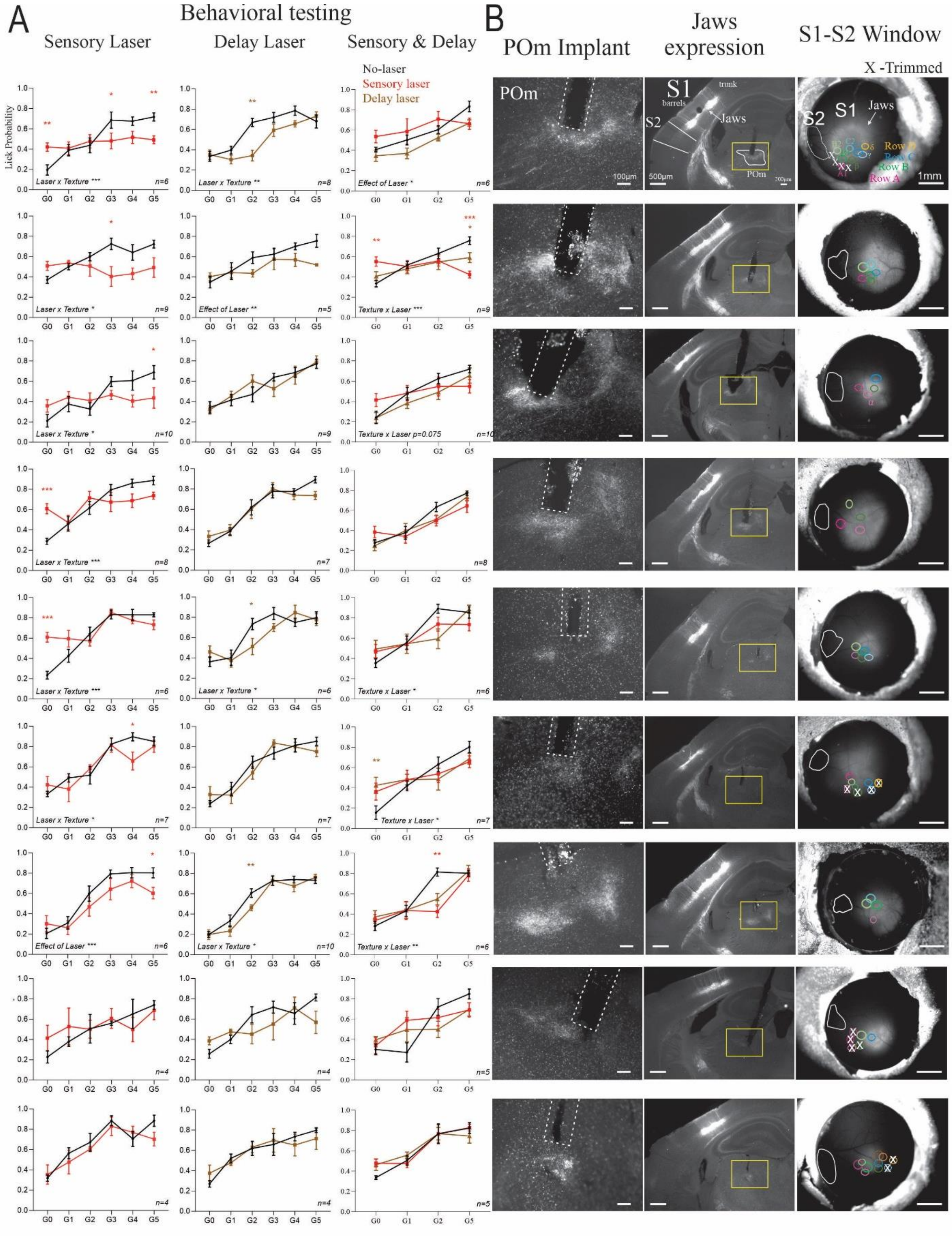
A
Behavioral testing
Sensory Laser
Delay Laser
Sensory & Delay
No-laser
Sensory laser
Delay laser
Lick Probability
Laser x Texture ***
Laser x Texture **
Effect of Laser *
Laser x Texture *
Effect of Laser **
Texture x Laser ***
Texture x Laser p=0.075
Laser x Texture *
Texture x Laser *
Texture x Laser **
Effect of Laser ***
G0 G1 G2 G3 G4 G5
B
POm Implant
Jaws expression
S1-S2 Window
X -Trimmed
POm
S1
barrels
trunk
S2
Jaws
100μm
500μm
1mm
Row D
Row C
Row B
Row A

Supplementary Figure 5. Discrimination performance and Jaws expression for individual mice

(**A**) Behavioral effects of sensory laser (left column), delay laser (middle column), and sensory and delay laser testing (right column), for Jaws-expressing mice. No-laser is represented by black, sensory laser by red and delay laser by brown lines. Each row are data from one mouse. Each graph indicates number of averaged sessions and significant 2-way RM ANOVA effects or interactions with Bonferroni post-hoc tests for cases of significant interactions. * $p<0.05$, ** $p<0.01$, *** $p<0.0001$. Values shown as mean ± standard error of the mean.

(**B**) Epifluorescence images of Jaws expression for the corresponding mice of each row in **A**. (Left column) Optic fiber implant locations in POm and (middle column) corresponding Jaws expression in S1 L5. (Right column) Images of Jaws-TdTomato viewed through the S1-S2 cranial window. Intrinsic signal imaging results are overlaid to identify S1 barrels and S2. 'X' over an S1 barrel indicates the associated whisker was trimmed down to the whisker pad due to an absence of Jaws expression.

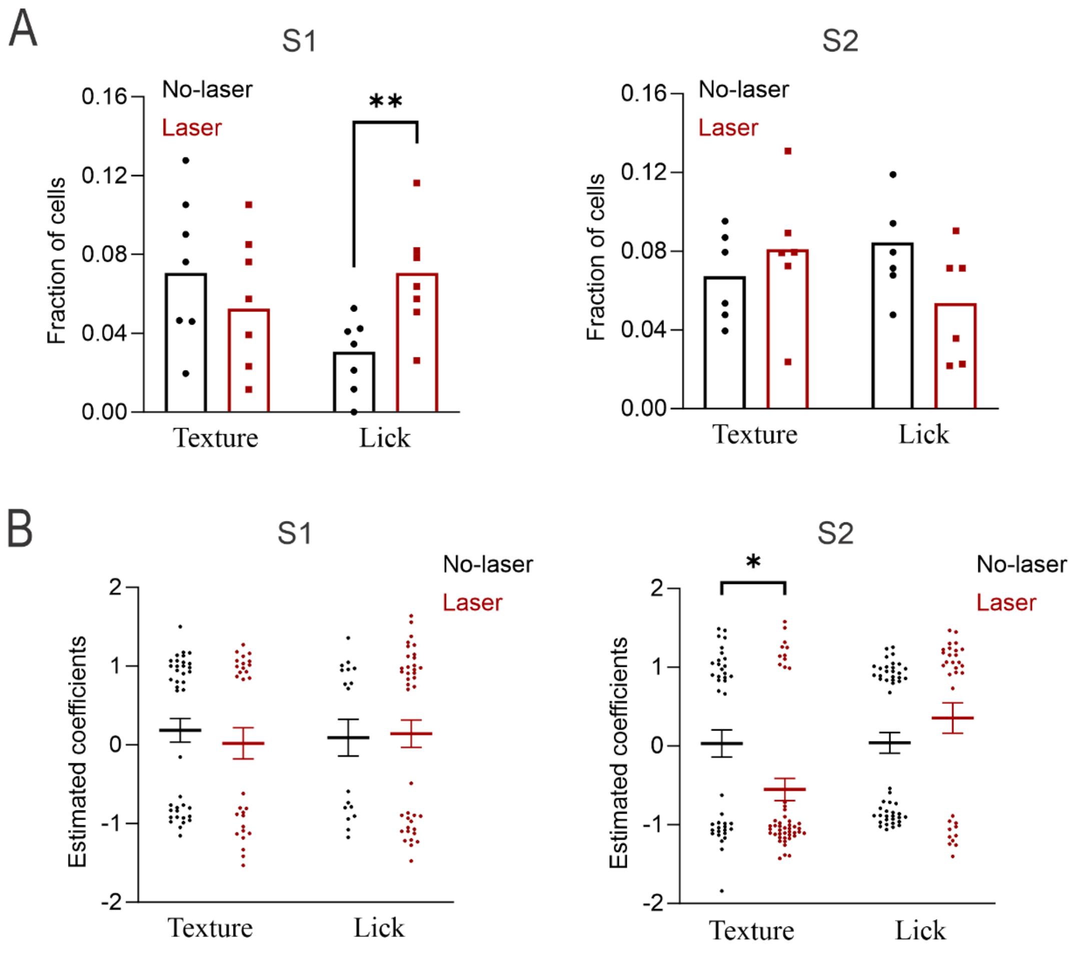

Supplementary Figure 6. Linear regression analysis to decode texture and choice variables in the response of single neurons during the sensory epoch

(**A**) Texture responsiveness of the calcium signal (AUC sensory - AUC baseline) was fitted to two categorical task-relevant predictors, texture and lick choice, using a linear model. Graphs show fractions of total population of recorded neurons with significant regression coefficients for each predictor. Bar graphs are averaged data across experiments and dots represent individual experiments. No-laser is represented by black, sensory laser by red. Laser inhibition increased the proportion of cells with significant regressors for lick choice in S1 cells. S1: 549 cells, 7 experiments, S2: 585 cells, 6 experiments. Z-tests, S1 Texture: no-laser vs laser, p=0.21, S1 Lick: no-laser vs laser, p=0.0025, S2 Texture: no-laser vs laser, p=0.32, S2 lick: no-laser vs laser, p=0.064. Significance of each regressor was determined by comparing to a shuffled distribution.

(**B**) Significant regression coefficients averaged across pooled S1 (left) and pooled S2 (right) neurons, for no-laser (black) and laser (red) conditions. Texture was coded as 1=G5 and 0=G0, Lick was coded as 1=lick, 0=no-lick. Laser inhibition inversed the relationship with the texture variable for S2 cells. Two-way ANOVA, S1: effect of variable p=0.94, effect of laser 0.77, variable x laser p=0.58. S2: effect of variable p=0.0045, effect of laser p=0.40, variable x laser p=0.0054, Bonferroni post-hoc for texture no-laser vs laser, p=0.017. S1 texture: n=39, S1 lick: n=17, S1 laser texture: n=29, S1 laser lick: n=39, S2 texture: n=40, S2 lick: n=50, S2 laser texture: n=48, S2 laser lick: n=32.

*p>0.05, ** p<0.01. Values shown as mean ± standard error of the mean.
